# Pulse Shape Discrimination of n/γ in Liquid Scintillator at PMT Nonlinear Region Using Artificial Neural Network Technique

**DOI:** 10.3390/s24248060

**Published:** 2024-12-18

**Authors:** Eungyu Yun, Ji Young Choi, Sang Yong Kim, Kyung Kwang Joo

**Affiliations:** Center for Precision Neutrino Research, Department of Physics, Chonnam National University, Gwangju 61186, Republic of Korea

**Keywords:** pulse shape discrimination, liquid scintillator, photomultiplier tube, artificial neural network, gamma and neutron separation, saturation, linearity

## Abstract

Reactor-emitted electron antineutrinos can be detected via the inverse beta decay reaction, which produces a characteristic signal: a two-fold coincidence between a prompt positron event and a delayed neutron capture event within a specific time frame. While liquid scintillators are widely used for detecting neutrinos reacting with matter, detection is difficult because of the low interaction of neutrinos. In particular, it is important to distinguish between neutron (n) and gamma (γ) signals. The principle of the interaction of neutrons with matter differs from that of gamma rays with matter, and hence the detection signal’s waveform is different. Conventionally, pulse shape discrimination (PSD) is used for n/γ separation. This study developed a machine learning method to see if it is more efficient than the traditional PSD method. The possibility of n/γ discrimination in the region beyond the linear response limits was also examined, by using 10- and 2-inch photomultiplier tubes (PMTs) simultaneously. To the best of our knowledge, no study has attempted PSD in a PMT nonlinear region using artificial neural networks. Our results indicate that the proposed method has the potential to distinguish between n and γ signals in a nonlinear region.

## 1. Introduction

Neutrino oscillation implies the existence of neutrino mass, which cannot be explained by the Standard Model. This suggests the existence of new physics beyond the Standard Model [1,2]. The interaction cross section of neutrinos is exceedingly small, as shown by the calculations of Bethe and Peierls [3]. Challenges faced in observing neutrino oscillations or detecting neutrinos have been addressed in two main ways: by increasing the amount of material used for detection and by significantly enhancing the number of interactions between neutrinos and the detection material [4,5,6].

Antineutrinos interact with protons through weak interactions, specifically inverse beta decay (${\bar{\nu }}_{e}+p\to e^{+}+n$), which produces a positron and a neutron. Through the detection of paired gamma rays emitted from the positron and neutron, neutrino events can be reconstructed.

Liquid scintillator (LS) detectors, which generate light upon absorbing radiation, are widely used in neutrino detection experiments [7,8,9,10,11]. Typically, the light emitted by the scintillator is converted to electrical signals by photomultiplier tubes (PMTs) and used for detection analysis [12,13]. While there are other options like silicon photomultipliers (SiPMs), PMTs were chosen for this application due to their ability to operate at room temperature with high gain and a large photo-detection coverage. This is critical for effectively converting the scintillator’s light output into electrical signals for the analysis.

In neutrino experiments, it is necessary to distinguish gamma rays generated by neutrino signals from background events caused by the surrounding environment. One of the background sources is cosmic rays, which can produce neutrons or gamma rays, that mimic neutrino signals [14]. Pulse shape discrimination (PSD) is used to differentiate between the two particles since they interact differently with the scintillator, resulting in slight variations in the detected signal waveforms [15,16,17].

Light signals generated by particle interactions in the scintillator are detected and amplified by PMTs. Although the total charge of the amplified signal should increase linearly with the amount of incoming light, it has been reported that when a large amount of light enters the PMT, the output signal becomes saturated, which distorts the waveform [18]. Investigating the limit of linearity and the behavior in the nonlinear region is essential for optimal signal reconstruction. Accordingly, several experiments have been conducted to examine the deviations from the linear performance of PMTs [19,20,21]. In this study, artificial neural networks (ANNs) were chosen for their ability to effectively learn and model the complex behavior and nonlinear relationships present in the PMT nonlinear region.

Antineutrinos interact with protons via weak interactions, but owing to the small scattering cross section, the detector size can be increased to improve reaction probability to a certain extent. Large-diameter PMTs are predominantly used to cover the scintillation detection area of large-scale detectors. ANNs were used to distinguish between neutron (n) and gamma-ray (γ) signals, particularly in the PMT nonlinear region of Hamamatsu’s 10-inch large-diameter PMT (R7081). ANNs are used in various domains of particle physics [22,23,24]. In particular, they are used in particle physics experiments. One of the main motivations for this study was to focus on this nonlinear region. The ANNs are known to be effective in solving nonlinear problems [25,26].

## 2. Experimental Setup

**Figure 1:**
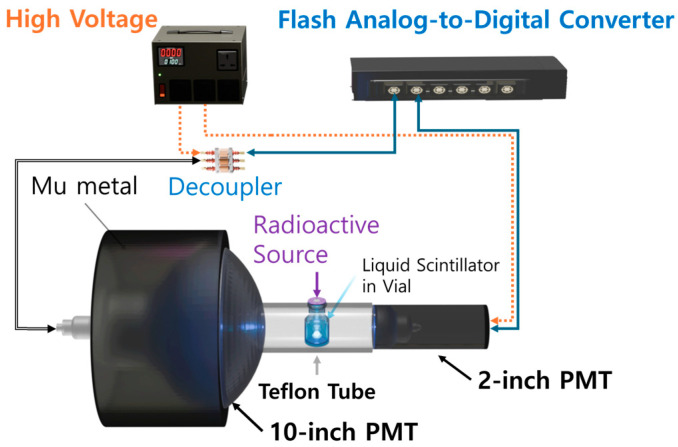
Experimental setup of 10-inch PMT (R7081) and 2-inch PMT (H7195) for measuring the scintillating lights from LS. The vial was placed at the center of a hollow Teflon tube with a length of 5.0 cm and a diameter of 2.1 cm. A ^252^Cf radioactive source with an activity of 46 kBq was placed in the vial.

An experiment was conducted to study the separation of n and γ signals in the LS on a laboratory scale, and the setup is illustrated in Figure 1. A 10 mL vial containing the LS was placed at the center of a Teflon tube, with two different PMTs positioned on either side.

An LS contains a mixture of an organic base solvent and fluor. In this study, diisopropylnaphthalene (C_16_H_20_) was used as the organic base solvent. This solvent is recognized for its suitability in PSD applications, and commercially available EJ-309 was used [27,28]. It has a flash point of 144 °C, reducing the risk of fire, while offering properties such as low vapor pressure, low toxicity, and compatibility with cast acrylic plastics [29]. In general, for the fluor 2,5-diphenyloxazole (C_15_H_11_NO) and 1,4-bis(2-methylstyryl)benzene ((CH_3_C_6_H_4_CH=CH)_2_C_6_H_4_) can be dissolved in an organic base solvent.

A Teflon tube held the vial in place and directed light from the scintillator to the PMTs. A 10-inch PMT (R7081) and a 2-inch PMT (H7195) were attached to the Teflon tube, with the 10-inch PMT shielded by μ-metal to exclude magnetic effects from the surroundings [30].

A ^252^Cf source was placed above the LS vial to emit neutrons and gamma rays, and signals from the PMTs were analyzed using a custom-built 400 MHz flash analog-to-digital converter (FADC) data acquisition (DAQ) system. The signal threshold for both PMTs was set to 6 mV, and data were acquired using a coincidence trigger. The gains for the 10-inch PMT and 2-inch PMTs were set to $1.06\times 10^{7}$ and $2.78\times 10^{6}$, respectively, to ensure that the PMT signal amplitudes remained within the dynamic range of the data acquisition (DAQ) system, which is from −2 V to 2 V. Attenuators (KN320, Kaizuworks Corporation, Tokyo, Japan) were installed to further adjust the signal levels and optimize the dynamic range of the DAQ electronics.

## 3. Measurements

**Figure 2:**
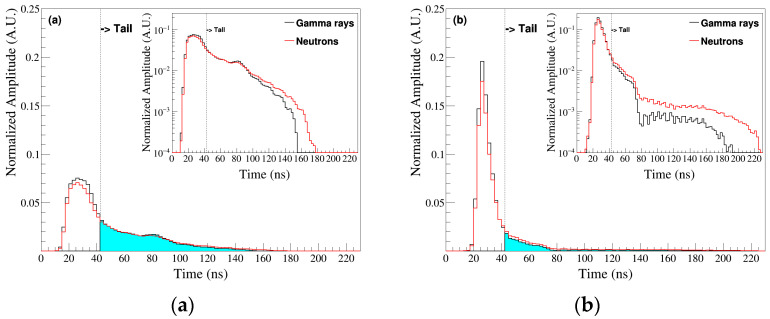
Examples of accumulated PMT signals for a photoelectron count range between 1500 and 1600: (**a**) the 10-inch PMT and (**b**) the 2-inch PMT (red: n signal, black: γ signal). The region highlighted in cyan represents the tail of the signal. (Inset: log scale). When viewed on a log scale, it is clearer that the neutron signal had a longer tail and thicker features than the gamma signal. Furthermore, there is a difference in pulse shape between the 10-inch PMT and 2-inch PMT.

Figure 2 shows examples of accumulated PMT signal shapes for n and γ signals between 1500 and 1600 photoelectrons (PEs). This PE range was specifically chosen because our analysis indicates that the PMT nonlinear response becomes prominent within this region, meaning that it is well suited for evaluating the performance of our nonlinear correction method. Neutrons lose energy through elastic collisions with nuclei, while gamma rays primarily interact with electrons, exciting them and causing light emission. Neutron signals, generated in multiple energy loss stages via strong interaction, have longer tails compared with γ signals, which are mainly generated through photoelectric or Compton scattering. Figure 2a shows the signals for the 10-inch PMT, and Figure 2b shows the signals for the 2-inch PMT. For both PMTs, n signals had broader tails than γ signals. This difference in tail shape is further accentuated by signal processing method in the DAQ, which utilizes a leading-edge discrimination technique with a threshold set above the noise floor (or pedestal) and a consistent synchronization delay (or without delay). The optimal upper (or lower) limit in time domain for PSD parameters, determined to be ~40 ns based on our analysis of the measurement dataset, effectively captures the distinct tail characteristics of neutron and gamma signals.

**Figure 3:**
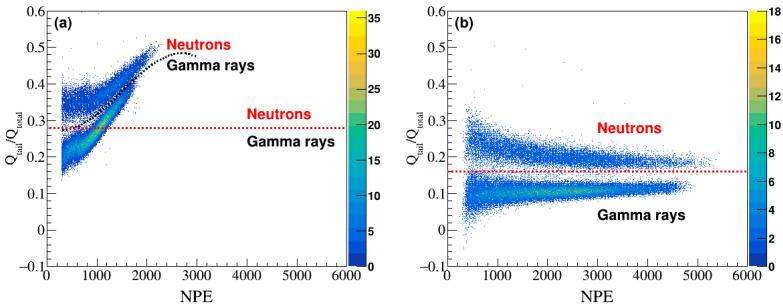
PSD scatter plots of Q_tail_/Q_total_ as a function of NPE for the (**a**) 10-inch PMT and (**b**) 2-inch PMT. Neutron and γ signals can be separated on the basis of the ratio Q_tail_/Q_total_ using red lines. The black dotted line in (**a**) indicates that the separation was performed using a polynomial method.

Particle identification was performed using a range of signal processing techniques, and the PSD method was regarded as an effective and reliable approach [31]. In PSD, charge comparison is assessed from the ratio of the total charge (Q_total_) to the tail charge (Q_tail_) to compare the differences in tail lengths between n and γ signals (Figure 2, tail region, colored cyan) [32]. The tail integration region is defined up to ~40 ns, which was determined to be optimal based on our analysis of measurement dataset. Figure 3 presents 2D scatter plots of the Q_tail_/Q_total_ ratio as a function of increasing the number of photoelectron (NPE) for the 10-inch and the 2-inch PMTs. Figure 3a,b show that n and γ signals separated around Q_tail_/Q_total_ = 0.28 (0.16) in the case of the 10-inch (2-inch) PMT. For the 2-inch PMT in Figure 3b, neutron signals, having longer tails, correspond to higher ratios (>0.16), while γ signals have lower ratios (<0.16). Unlike the 2-inch PMT, the 10-inch PMT (Figure 3a) shows a nonlinear relationship between the Q_tail_/Q_total_ ratio and NPE. The commonly used polynomial method was implemented and indicated by a black dotted line in Figure 3a. While a simpler approach like fitting a polynomial curve to the data in Figure 3a might appear sufficient for separating the neutron and gamma signals, the inherent nonlinearities within the data, such as gain saturation, necessitate a more sophisticated method. Furthermore, the ANN’s ability to analyze and classify data in high-dimensional space highlights its effectiveness in handling complex data structures. By effectively learning these nonlinearities, the ANN achieves improved classification results.

**Figure 4:**
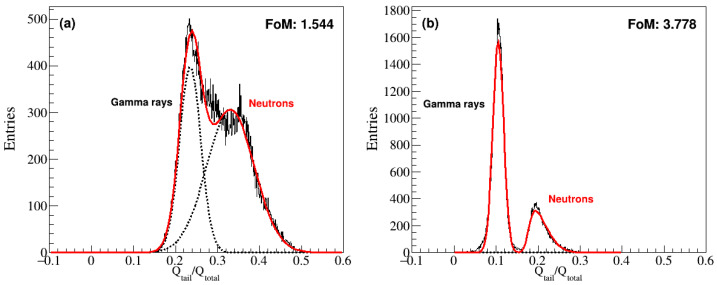
Number of events—Q_tail_/Q_total_ for the (**a**) 10-inch PMT, where the FoM value is obtained from two Gaussian peaks indicated from n and γ signals; (**b**) 2-inch PMT, where a skew-Gaussian peak is applied to the n signals.

Figure 4 projects the scatter plot in Figure 3 along the y-axis, and two Gaussian peaks corresponding to n and γ signals can be observed. By utilizing an ANN, we effectively map the data into a higher-dimensional space where a linear decision boundary can successfully separate the neutron and gamma signals. In general, a figure of merit (FoM) is defined as a quantity or parameter introduced for characterizing the performance of a system or algorithm. In our study, the capacity to distinguish between n and γ signals represented the performance evaluation factor of the algorithm in question. The PSD evaluation factor (or FoM) is defined as

$$FoM=\frac{\Delta S}{\sqrt{\sigma_{x}^{2}+\sigma_{y}^{2}}},$$

where ΔS represents the absolute difference in the means of the two distributions, and $\sigma_{x}^{2}$ and $\sigma_{y}^{2}$ are the variances of the respective peaks [33]. To account for the asymmetry in n signal distribution, a skew–Gaussian distribution was applied. This distribution exhibited a moderate skew to the right from the normal distribution. For consistency, the FoM was redefined with a simple substitution of the mean and variance derived from the skew–Gaussian fit. Figure 4a shows overlapping peaks because of the nonlinear relationship between Q_tail_/Q_total_ and NPE in the 10-inch PMT, and they result in a low FoM of 1.552. In contrast, the 2-inch PMT signals in Figure 4b have well-separated peaks and a higher FoM of 3.778.

## 4. ANN Training

In this study, after obtaining nonlinear results with the traditional PSD method, we converted the hierarchical and nonlinear data formats used in high-energy physics into a machine learning development environment based on the PyTorch (2.0.1 version) framework [34]. An experiment was performed by using the ANN to enhance the PSD performance in the PMT nonlinear region of the 10-inch PMT. The 2-inch PMT signals, which showed high FoM, were used as labels, and the 10-inch PMT signals in the region were used as data for the ANN training.

**Figure 5:**
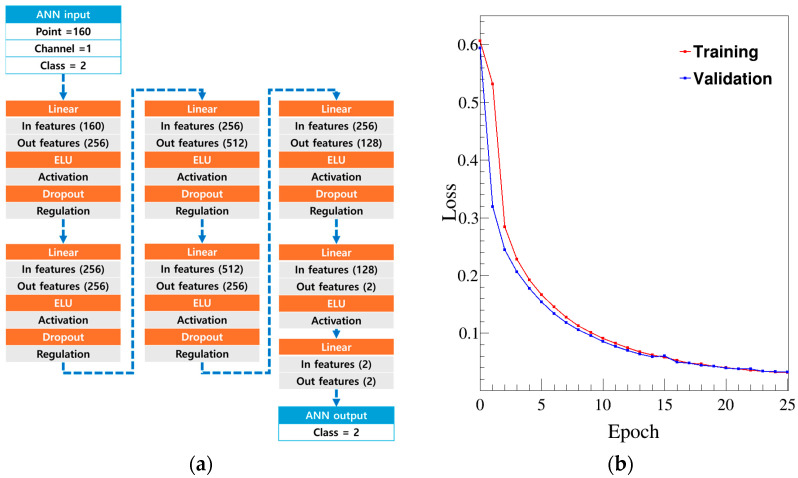
Structure and training of the ANN: (**a**) a schematic of the ANN used to separate n and γ signals from the observed pulse shape; (**b**) decrease in loss of the training and validation sets during the epoch.

To ensure the accuracy of the final results, we used 40% of the total experimental data for training, 30% for validation, and 30% for testing. Figure 5a shows the neural network structure. The input layer consisted of 160 nodes; this value was determined by dividing the optimized time domain of 400 ns by a signal acquisition interval of 2.5 ns. The signal was then passed through weights connecting each node in the linear regression layer to the linear regression hidden layer, rather than through the convolution layer, and it was processed using an exponential linear unit activation function before being passed to subsequent layers [35]. The signal passed through several intermediate layers before reaching the output layer, where it was classified into n and γ signals, as shown in Figure 5a.

Figure 5b shows the training process in each epoch. The optimization method employed was a first-order gradient-based stochastic optimization technique, specifically adaptive moment estimation [36]. To prevent overfitting, we used a dropout method. The loss function was a binary entropy loss function that could differentiate between neutron and gamma ray signals, since the classification was binary. As training progressed, the loss value decreased, and the validation value, used to verify that training was proceeding correctly, also steadily decreased. The loss value reached its minimum around epoch 25. The trained ANN was tested to evaluate its classification accuracy with data that were not used for training and validation.

## 5. Results

**Figure 6:**
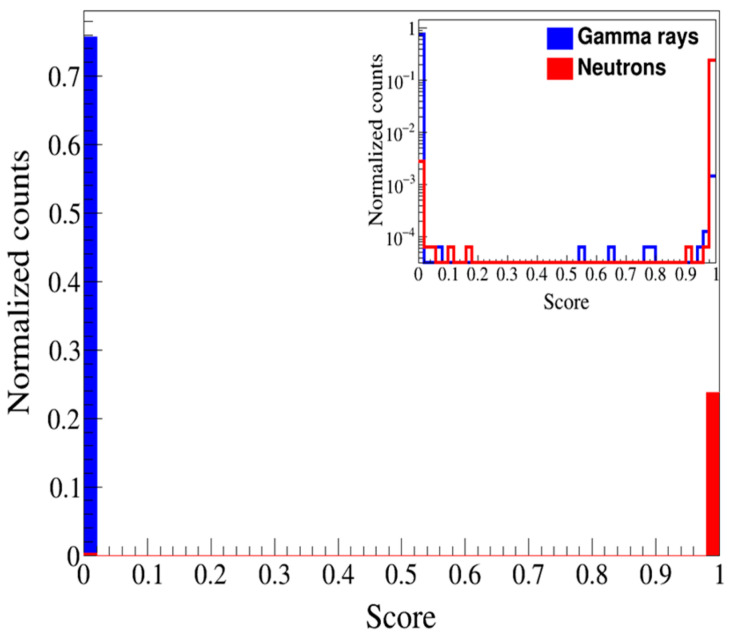
ANN score graph of n and γ signals (inset: log scale). If the score value was above 0.5, it was closer to neutrons, and if it was below 0.5, it was closer to gamma rays.

Figure 6 shows the ANN score values, which were based on probabilities derived from the SoftMax function that was applied to the test data [37,38]. The ANN classified signals as neutrons if their value was greater than 0.5 and as gamma rays for all other values. In Figure 6, neutrons are indicated in red and gamma rays in blue on the basis of the labels. When compared with the labels, accurate classification by the ANN corresponds to cases where the red value exceeds 0.5 or the blue value is less than 0.5. Applying the traditional linear threshold of Q_tail_/Q_total_ (=0.28) value to the 10-inch PMT signals resulted in an accuracy of 70.7% when compared with the 2-inch PMT reference results, the accuracy of the polynomial method was 99.4% (Figure 3a). The ANN achieved a classification accuracy of 99.5%. The Gaussian mixed models and probabilistic support vector machines approaches yielded FOM values of around 3, while the Boosted Decision Trees algorithm-based statistical learning approach yielded similar results [39,40].

## 6. Summary

There is a slight difference between the detection signal waveforms of neutrons and gamma rays, and generally a PSD method can be used to differentiate them. In this study, the nonlinear behavior of a PMT was investigated by examining PMT saturation responses, and the distortion of the pulse shape. We aimed to enhance the PSD performance by employing an ANN to differentiate between neutrons and gamma rays in the PMT nonlinear region. Our results showed that the polynomial method (ANN) showed 99.4% (99.5%) discrimination capability. The ANN is capable of automation, and adaptable to diverse datasets when sufficient data and reliable labels are available.

By using two PMTs receiving signals from identical events, the research validated the method’s efficacy through experimental observations, without presuming that its functionality in the nonlinear region would be the same as that in normal conditions. This approach allows us to extract important features from one-dimensional time series data using a straightforward combination of linear regression layers. This method is effective even when the PMT response is nonlinear, making it a versatile tool for data analysis.

Notably, this study was conducted using experimental data, not simulations, and n and γ signals were directly used without taking recourse to other particles known to interact similarly with LSs. This approach effectively addresses concerns about whether the labels accurately reflected real experimental conditions. However, the possibility of the experimental uncertainty affecting the ANN performance was noted, as the use of labels generated from experimental data introduces a degree of uncertainty into the training process. The inherent uncertainty may affect the accuracy of the ANN, and its performance could vary with PMT models or experimental setup. Overall, this study shows that the sensitivity of PMT-based detection systems in particle physics experiments can be potentially enhanced. The successful application of the ANN to distorted signals opens new avenues for its use in other fields requiring precise signal discrimination and analysis.

## Data Availability

Data are contained within the article.
